# Disruption of Autophagic Flux and Treatment with the PDPK1 Inhibitor GSK2334470 Synergistically Inhibit Renal Cell Carcinoma Pathogenesis

**DOI:** 10.7150/jca.92521

**Published:** 2024-01-20

**Authors:** Weimin Zhou, Ji Huang, Chuansheng Huang, Gongxian Wang, Xinhua Tu

**Affiliations:** 1 Department of Urology, Jiangxi Cancer Hospital, The Second Affiliated Hospital of Nanchang Medical College, Jiangxi Clinical Research Center for Cancer, Nanchang, Jiangxi 330000, China.; 2 Department of Pathology, Jiangxi Cancer Hospital, The Second Affiliated Hospital of Nanchang Medical College, Jiangxi Clinical Research Center for Cancer, Nanchang, Jiangxi 330000, China.; 3 Department of Urology, The First Affiliated Hospital of Nanchang University, Nanchang, Jiangxi 330000, China.

**Keywords:** Renal cell carcinoma, 3-Phosphoinositide-dependent kinase 1, Autophagy, PI3K-Akt-mTOR pathway, Targeted therapy

## Abstract

**Background:** Renal cell carcinoma (RCC) frequently exhibits activating PI3K-Akt-mTOR pathway mutations. 3-Phosphoinositide-dependent kinase 1 (PDPK1 or PDK1) has been established to play a pivotal role in modulating PI3K pathway signaling. mTOR is the main autophagy-initiating factor. However, limited advances have been made in understanding the relationship between PDPK1 and autophagy in RCC.

**Methods:** GSK2334470 (GSK470), a novel and highly specific inhibitor of PDPK1, was selected to investigate the anticancer effects in two RCC cell lines. Cell growth was assessed by CCK-8 test and colony formation. Changes in the protein levels of key Akt/mTOR pathway components and apoptosis markers were assessed by Western blotting. Autophagy was assessed by using LC3B expression, transmission electron microscopy, and a tandem mRFP-EGFP-LC3 construct. The effect of PDPK1 and autophagy inhibitor chloroquine in RCC *in vivo* was examined in a mouse tumor-bearing model.

**Results:** GSK470 significantly inhibited cell proliferation and induces apoptosis in A498 and 786-O RCC cells. GSK470 downregulates the phosphorylation of PDPK1, thereby inhibiting downstream phosphorylation of Akt1 at Thr308 and Ser473 and mTOR complex 1 (mTORC1) activity. Treatment with insulin-like growth factor-1 (IGF-1) partially restored GSK470-induced behaviors/activities. Interestingly, treatment of A498 and 786-O cells with GSK470 or siPDPK1 induced significant increases in the hallmarks of autophagy, including autophagosome accumulation, autophagic flux, and LC3B expression. Importantly, GSK470 and chloroquine synergistically inhibited the growth of RCC cells *in vitro* and in xenograft models, supporting the protective role of autophagy activation upon blockade of the PDPK1-Akt-mTOR signaling pathway.

**Conclusion:** Our study provides new insight into PDPK1 inhibition combined with autophagy inhibition as a useful treatment strategy for RCC.

## Introduction

Renal cell carcinoma (RCC) accounts for approximately 4.1% of all new cancer cases 1. The incidence of RCC continues to increase in both men and women. The most common subtype is clear cell renal carcinoma (ccRCC), which accounts for approximately 70-80% of cases 1, 2. It is estimated that approximately 25% of RCC patients who seek treatment already have locally advanced disease, lymph node metastasis or distant metastasis, and 20% of RCC patients who undergo radical surgery will later experience local recurrence or distant metastasis 3. In-depth research on the molecular mechanisms underlying the occurrence and development of RCC has facilitated the use of therapeutic approaches such as tyrosine kinase inhibitors (TKIs) or immunotherapy to treat metastatic ccRCC. However, the effective durations of TKI therapy and immunotherapy alone and in combination remain limited, and drug resistance inevitably emerges; thus, the 5-year survival rate remains lower than 10% 4.

The PI3K/Akt/mTOR pathway is a signaling pathway that plays a key role in regulating cell growth, proliferation, and survival 5-7. In RCC, the PI3K/Akt/mTOR pathway is frequently activated due to genetic mutations and aberrant signaling 8. Thus, targeting the PI3K/Akt/mTOR pathway has emerged as a promising therapeutic strategy for RCC. Several inhibitors of this pathway, such as mTOR inhibitors (rapamycin, everolimus, and temsirolimus), exhibit substantial antitumor activity and improve progression-free survival (PFS) in patients with advanced RCC. However, their clinical benefits are limited by the development of resistance, which is believed to be related to autophagy induced by mTOR inhibition.

3-Phosphoinositide-dependent kinase 1 (PDPK1 or PDK1) functions downstream of PI3K and is crucial for the activation of Akt and many other AGC kinases 9, 10. Accumulating evidence has highlighted PDPK1 as an intriguing and underexplored target for cancer therapy 7. Herein, we investigated the anticancer effect and the alterations in the PDPK1-Akt-mTOR signaling cascade upon pharmacological inhibition of PDPK1 with GSK2334470 (GSK470). Additionally, the relationship between autophagy and PDPK1 inhibition was systematically studied.

## Materials and Methods

### Cells and cell culture

Two RCC cell lines 786-O and A498, and one normal human renal tubular epithelial cell line HK-2 were obtained from Procell Life Science Company (Wuhan, China). A498 and HK-2 cells were cultured with MEM (Solarbio, Science & Technology, Beijing, China), and 786-O cells were cultured with RPMI-1640 medium (Solarbio Science & Technology, Beijing, China); both media were supplemented with 10% FBS (Biological Industries, Beit Haemek, Israel), 100 units/ml penicillin and 100 µg/ml streptomycin, and incubation was carried out at 37 °C in 5% CO_2_ and saturated humidity. Cells were authenticated by short tandem repeat profiling.

### siRNA-mediated gene silencing

The siRNAs for targeted knockdown of *ATG5* (Cat# sc-41445) and *ATG7* (Cat# sc-41447) and the control siRNA (Cat# sc-44232) were purchased from Santa Cruz Biotechnology (Texas, USA). The siRNA targeting the PDPK1 gene (siPDPK) and the corresponding scrambled siRNA (siControl) were synthesized by Hanheng Biotechnology (Wuhan, China). These siRNA sequences were as follows: siPDPK—sense sequence: CGACGAGGACUGCUAUGGCAAUUAU; antisense sequence: AUAAUUGCCAUAGCAGUCCUCGUCG. siControl—sense sequence: CGAAGGACGUCGUAUAACGUGCUAU; antisense sequence: AUAGCACGUUAUACGACGUCCUUCG. RCC cells were transfected with 2 μg/mL targeted siRNA or control siRNA mixed with Lipofectamine 3000 transfection reagent (Thermo Fisher, Waltham,MA, USA) according to the manufacturer's instructions. After 6 h, the Opti-MEM was replaced by medium supplemented with 10% FBS. The silencing efficiency was determined by RT‒qPCR and immunoblotting.

### Chemicals and reagents

Reagents were obtained as follows. GSK470 (S7087), chloroquine (CQ) (S6999) and sunitinib malate (S1042) were obtained from Selleck (Houston, TX, USA). IGF-1 (CA32029) was obtained from Cellapy (Beijing, China). Antibodies specific for p-PDPK1, PDPK1, Akt, p-Akt (Thr308), pAkt (Ser473), mTOR, p-mTOR (Ser2448), p70S6K, p-p70S6K (Thr389), 4EBP, ATG5, and ATG7 were obtained from Cell Signaling Technology (Danvers, MA, USA). The anti-Ki67 antibody (GB111141), anti-GAPDH antibody (GB11002) and goat anti-rabbit horseradish peroxidase (HRP)-conjugated secondary antibody (GB23303) were obtained from Servicebio Technology (Wuhan, China). The GFP-mRFP-LC3 adenovirus (#HB-AP210) was purchased from Hanheng Biotechnology (Shanghai, China).

### Cell proliferation and colony formation assays

A Cell Counting Kit-8 (CCK-8) assay (#BS350B, Biosharp, Hefei, China) was applied to evaluate cell proliferation. Cells (2× 10^3^ cells/well) were cultured in 96-well plates. At the end of the experiment, a mixture of CCK-8 solution and cell culture medium (1:9) was added to the 96-well plates, which were incubated at 37 °C for 2 h while shielded from light. Negative control wells containing medium and CCK-8 reagent but no cells were also set up. The OD value of each well at a wavelength of 450 nm was measured using a SpectraMax 190 instrument (Molecular Devices, CA, USA) and corrected on the basis of the OD value of the negative control group. To assess colony formation, 300 cells were seeded in each well of a 6-well plate and maintained in medium containing 10% FBS at 37 °C. The medium was replaced every three days. After 14 days, colonies were fixed with methanol and stained with 0.1% crystal violet (Solarbio, Science & Technology, Beijing, China). Visible colonies were manually counted, and triplicate measurements were performed for each treatment group.

### Transmission electron microscopy

Transmission electron microscopy of cells was performed as previously described 9. RCC cells were treated with GSK470 (4 μM) for 24 h and harvested for imaging. In brief, sections were sliced with a UC-7 Ultracut microtome (Leica), stained with uranyl acetate and lead citrate, and examined in a JEM-1400 transmission electron microscope (Japan Electron Optics Laboratory Co., Ltd., Tokyo, Japan). At a voltage of 80 kV, the slice was located at low magnification with the fluorescent screen, a CCD was inserted (camera model: MORADA; brand: EMSIS; image acquisition and measurement software: RADIUS ALL 2.2, Build 21230), the target structure was visualized, the electron microscope was adjusted until the image was clear, and the required image was acquired.

### Assessment of autophagic flux

Autophagic flux was measured using the mRFP-GFP-LC3 adenovirus (#HB-AP210) purchased from Hanheng Biotechnology (Wuhan, China) as previously described. This assay is based on the differences in the pH stability of green and red fluorescent proteins 10. The fluorescence signal of GFP can be quenched under the acidic conditions (pH<5) in the lysosome, but the mRFP fluorescence signal does not significantly change under acidic conditions. In the merged green and red fluorescence images, autophagosomes are visible as yellow puncta (RFP+GFP+), while autolysosomes are visible as red puncta (RFP+GFP-). Autophagic flux is increased when the numbers of both yellow and red puncta are increased in cells. RCC cells were infected with the mRFP-GFP-LC3 adenovirus for 48 h and were then treated with or without GSK470 (4 μM) for 24 h. The formation of autolysosomes was detected and analyzed using a confocal microscope (Leica STELLARIS 5).

### RT-qPCR

Total RNA was extracted from cells using TRIzol reagent (Takara, Japan). First-strand cDNA synthesis was performed with 1 μg of RNA in a 20-μl reaction mixture using the High-Capacity cDNA Reverse Transcription Kit (Applied Biosystems, CA, USA). The *ATG5*, *ATG7*, *PDPK1* and *GAPDH* transcripts were amplified, and relative gene expression was calculated with the 2^-ΔΔ Ct^ method using *GAPDH* as a housekeeping gene. The amplification procedure was implemented as follows: 5 min of initial denaturation at 95 °C, then 40 cycles covering 10 s at 95 °°C, 30 s at 60 °C, and 30 s at 72 °C. Data were obtained from at least three independent experiments. The primer sequences used in this study are listed in Table 1.

### Flow cytometry

786-O and A498 cells were pretreated with DMSO or CQ (10 μM) for 4 h, treated with or without GSK470 (2 μM) for 48 h, harvested, added to 1 ml of precooled phosphate-buffered saline (PBS), and centrifuged at 1000 × g. The Annexin V/FITC (#1062 L) double-staining cell apoptosis detection kit (Beyotime, Shanghai, China) was used according to the manufacturer's instructions. The data were acquired and analyzed by flow cytometry and the associated software (Beckman Coulter, CA, USA).

### Immunoblotting

After treatment, cells were washed three times with PBS and homogenized in protein lysis buffer containing 10% phosphatase inhibitors. Cell debris was removed by centrifugation at 12,000 × g for 15 min at 4 °C. The supernatant (cell lysate) was used for immunoblotting. The protein concentrations in cell lysates were measured using a Bio-Rad protein assay kit (Thermo Scientific, Waltham,MA, USA). The proteins (20-70 µg) in each sample were separated by SDS‒PAGE and transferred to PVDF membranes (Millipore, Bedford, MA, USA), and the membranes were blocked for 1.5 h with 5% nonfat milk in Tris-buffered saline solution containing 0.1% Tween 20. The membranes were then incubated overnight at 4 °C with primary antibodies, washed, and incubated with HRP-conjugated species-specific secondary antibodies. Immunoreactions were detected by enhanced chemiluminescence (GLPBIO, CA, USA). All experiments were repeated at least three times.

### *In vivo* evaluation of the effects of GSK470 and CQ

786-O RCC cells (5 x 10^7^) were suspended in a mixture of HBSS and Matrigel and were then subcutaneously implanted into male mice (NOG background). Tumors were allowed to grow to a volume of 100 mm^3^ prior to treatment. Then, animals bearing xenografts derived from each cell line were randomized into 5 treatment groups consisting of 6 animals each. The mice were treated with vehicle (normal saline, ip, tiw), GSK470 (100 mg/kg, ip, tiw), CQ (65 mg/kg, ip, qd), sunitinib (80 mg/kg, po, 5d/w), or a combination of GSK470 and CQ for 4 weeks. The mice were monitored daily, and tumor volumes were measured twice weekly. At study completion, mice were sacrificed by CO_2_ asphyxiation, and tissues were collected, fixed with formalin, and embedded in paraffin for immunohistochemical analysis.

### Immunohistochemical analysis

Tumors were fixed with 4% paraformaldehyde, embedded in paraffin, and cut into 4-μm sections prior to dewaxing, rehydration, and sequential treatment with 0.01 M sodium citrate (pH 6.0), 3% hydrogen peroxide, and 1% goat serum with 0.2% Triton X-100. After incubation with a primary antibody, the slides were stained with HRP-conjugated goat anti-rabbit IgG H&L and visualized by 3,3-diaminobenzidine staining. Images were captured using a Leica fluorescence microscope and a 20X objective. ImageJ software was used to quantify expression by densitometric analysis of five random fields containing viable tumor cells. Quantification of Ki67 staining was conducted by counting the positive cells in five random fields.

### Statistical analysis

SPSS 21.0 and GraphPad Prism 8 software were used for statistical analysis of the experimental data. The statistical significance of differences between samples was determined using unpaired Student's *t* test*.* Differences among multiple groups were assessed by one-way ANOVA with Tukey post-hoc test. In all experiments, differences with a two-tailed *P* value of < 0.05 were considered significant. For all tests, * indicates that the difference between the two groups was statistically significant (*P < 0.05, **P < 0.01, ***P < 0.001).

## Results

### The PDPK1 inhibitor GSK470 suppresses RCC cell growth and induces apoptosis

**Figure 1:**
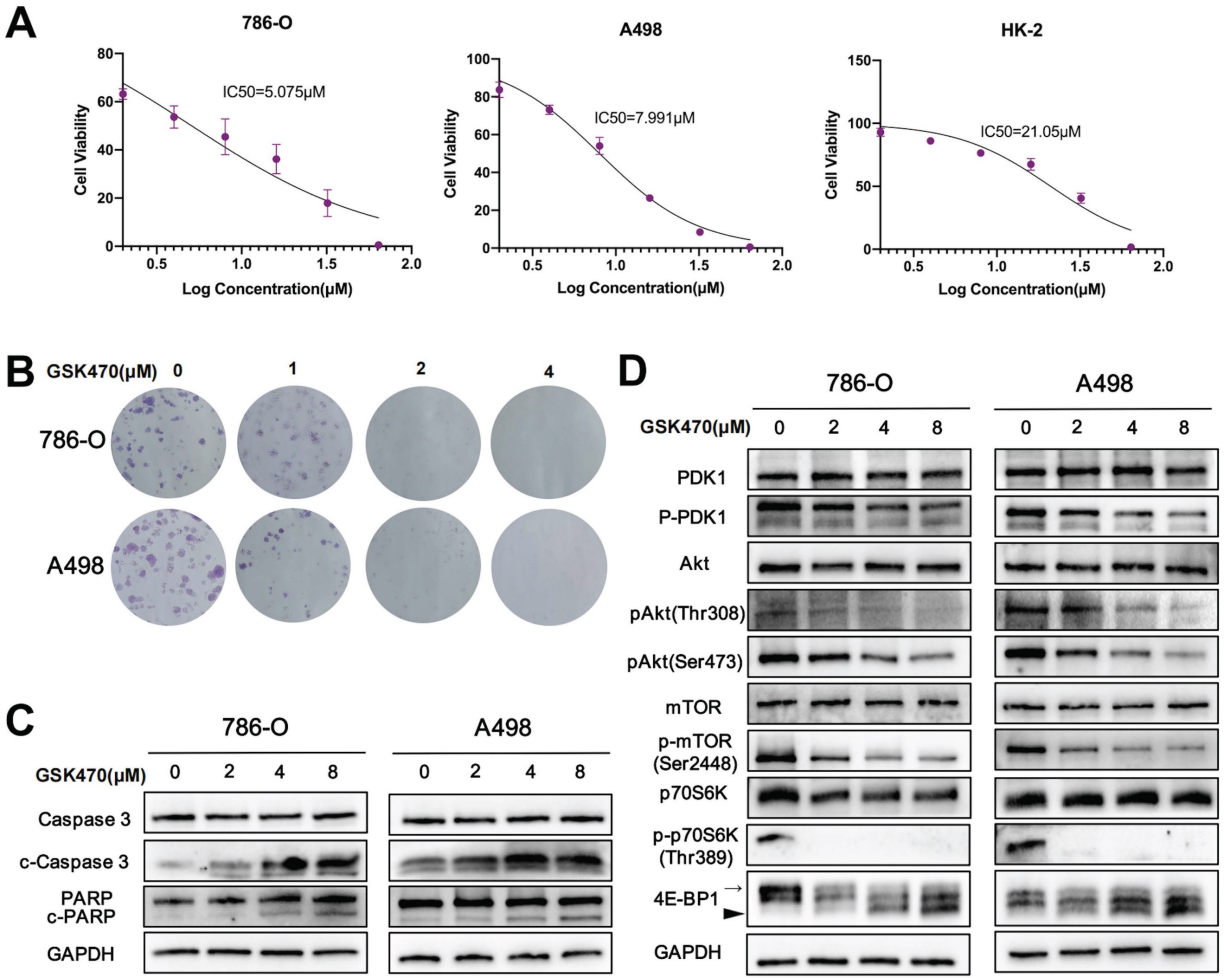
The PDPK1 inhibitor GSK470 inhibits RCC cell proliferation and induces apoptosis. **A.** Effects of GSK470 on the proliferation of A498 and 786-O cells. Cells were treated with 0.1% DMSO or GSK470 for 48 h, and cell viability was evaluated using a CCK-8 kit. **B.** Effect of GSK470 on RCC cell colony formation. RCC cells were treated with the indicated concentrations of GSK470 for 14 d and were then fixed, stained, and counted. Colony collection is shown on the left, and the colony number used for statistical analysis is shown on the right. **C.** Western blot analysis showing the effects of GSK470 on the levels of apoptosis-related proteins. A498 and 786-O cells were treated with GSK470 at the specified concentrations for 48 h. GAPDH was used as a loading control. **D.** Western blot analysis was performed to assess the expression of components of the PDPK1-Akt-mTOR pathway after treatment with GSK470 for 48 h. The thin and thick arrow indicates the hyperphosphorylated and hypophosphorylated form of 4E-BP1, respectively. The relative quantification of 4E-BP1 refers to the ratio of hyperphysiologylated form to hyperphysiologylated form. Mean ± SD, n = 3. * indicates a significant difference compared with the control group.

To assess the potential of PDPK1 as an anti-RCC target, we investigated the anticancer effects of the PDPK1 inhibitor GSK470 in two RCC cell lines and one normal renal cell line. The cells were treated with varying concentrations of GSK470, and cell viability was assessed. Our results demonstrated that GSK470 (0-64 μM) effectively inhibited the growth of 786-O and A498 RCC cells in a dose-dependent manner (Fig. 1A), with IC50 values of 5.075±1.51 μM and 7.991±0.57 μM, respectively. In contrast, the IC50 value in HK-2 cells was significantly higher (21.05±1.60 μM). Furthermore, the results of the colony formation assay showed that GSK470 inhibited colony formation by 786-O and A498 cells in a dose-dependent manner; even a low concentration (1 μM) of GSK470 significantly suppressed colony formation (Fig. 1B). We then performed immunoblot analysis of apoptotic markers to evaluate whether GSK470 inhibits growth by inducing apoptosis. Our results demonstrated that GSK470 effectively induced the cleavage of Caspase3 and poly ADP-ribose polymerase (PARP) in a dose-dependent manner (Fig. 1C), indicating that GSK470 triggers apoptosis in RCC cells. These *in vitro* findings suggested that targeting PDPK1 could be a promising therapeutic approach for RCC.

### GSK470 inhibits tumor growth by suppressing the PDPK1/Akt/mTOR signaling pathway

The PI3K/Akt/mTOR signaling pathway plays a crucial role in regulating malignant behaviors of RCC cells 11. PDPK1 has been reported to be involved in the PI3K/Akt signaling pathway 7. To investigate the cascading effects of GSK470-mediated targeting of PDPK1 in RCC, we performed immunoblotting to measure the expression of PDPK1, Akt, mTOR, and downstream members of this pathway. The p-PDPK1/PDPK1, p-Akt (Ser473)/Akt, and p-Akt (Thr308)/Akt ratios were decreased in a dose-dependent manner in both A498 and 786-O cells after treatment with GSK470 (Fig. 1D). Additionally, GSK470 treatment strongly inhibited the phosphorylation of mTOR at Ser2448, a marker for mTOR complex 1 (mTORC1) activity (Fig. 1D), consistent with previous studies 12, 13. mTORC1 can directly phosphorylate the translational regulators eukaryotic translation initiation factor 4E (eIF4E)-binding protein 1 (4E-BP1) and p70 ribosomal protein S6 kinase (p70S6K), which promotes protein synthesis, cell growth, and survival 14. Therefore, we evaluated changes in the phosphorylation of these two key regulators after treatment with GSK470. Hyperphosphorylation of 4EBP was successively decreased with increasing concentrations of GSK470 (Fig. 1D). Additionally, the phosphorylation of p70S6K (Thr389) was completely inhibited even after treatment with GSK470 at the lowest tested concentration (Fig. 1D).

**Figure 2:**
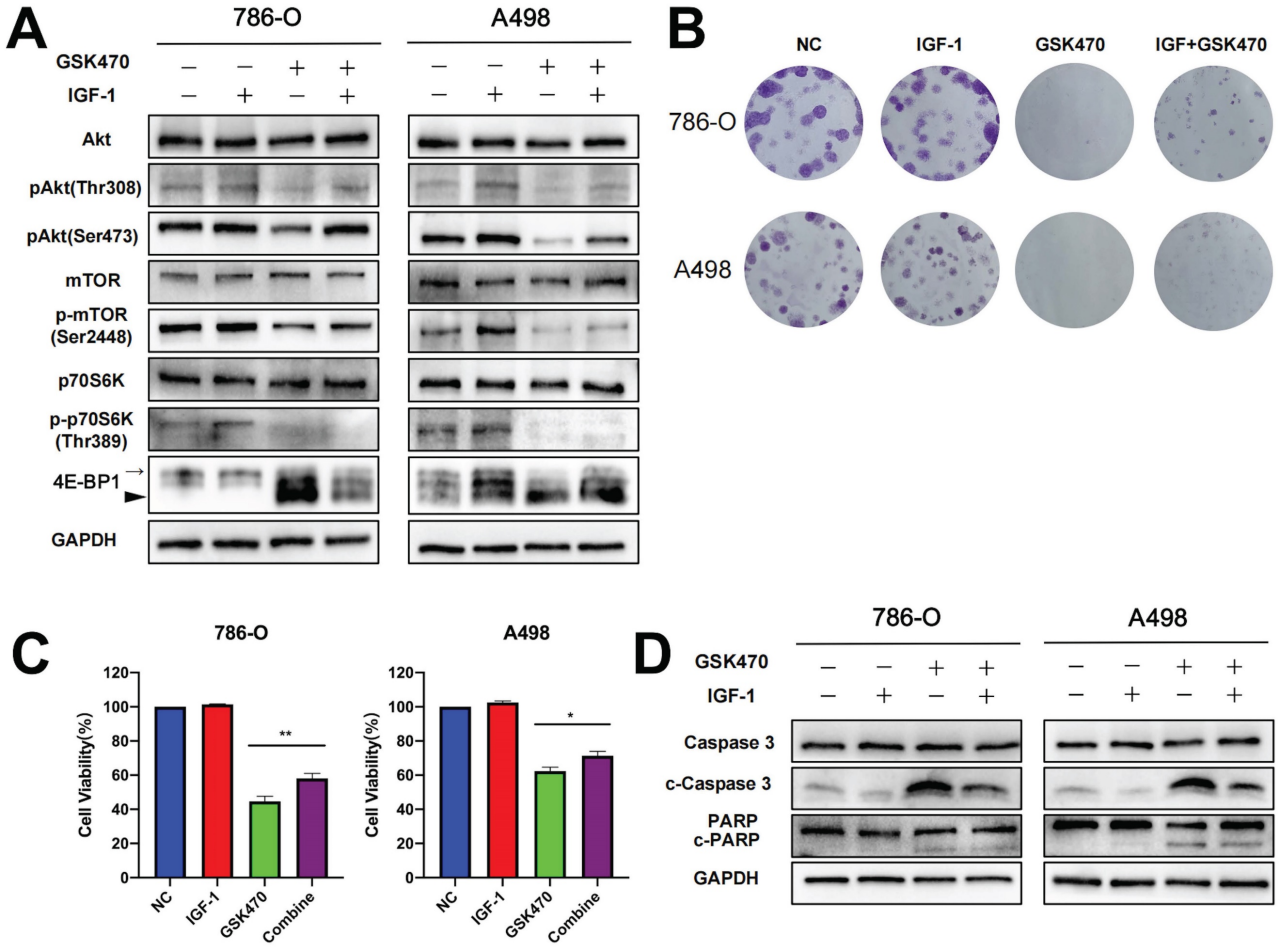
The PI3K/Akt agonist IGF-1 partially reversed the inhibition of the PDPK1/AKT/mTOR pathway, colony formation and apoptosis induced by GSK470. **A.** Western blot analysis showing the efficiency of IGF-1 in abrogating the effects of GSK470 on the PDPK1/AKT/mTOR pathway. RCC cells were incubated with or without 100 ng/ml IGF-1 for 2 h and were then cocultured with or without 4 μM GSK470 for 48 h. **B.** Effect of IGF-1 on changes in colony formation induced by GSK470. RCC cells were treated with DMSO, IGF-1 (100 ng/ml), GSK470 (4 μM), or a combination of IGF-1 and GSK470 for 14 d and were then fixed, stained, and counted. **C.** Effects of IGF-1 on changes in cell viability induced by GSK470 in RCC cells. Cells were treated with DMSO, IGF-1 (100 ng/ml), GSK470 (4 μM), or a combination of IGF-1 and GSK470, and cell viability was evaluated by a CCK-8 assay. **D.** Western blot showing the effect of IGF-1 on abrogating the change in apoptosis caused by GSK470. RCC cells were incubated with or without 100 ng/ml IGF-1 for 2 h and were then cocultured with or without 4 μM GSK470 for 48 h. Mean ± SD, n = 3. * indicates a significant difference between the indicated groups.

To investigate whether PI3K-Akt pathway activation can reverse the effect of GSK470 treatment, we pretreated cells with IGF-1, a well-known activator of the PI3K-Akt signaling pathway 15. Our results showed that the levels of phosphorylated Akt, mTOR, and 4EBP, which were reduced following exposure to GSK470, were restored upon pretreatment with IGF-1 (Fig. 2A). Interestingly, the dephosphorylation of p70S6K was not reversed by IGF-1 pretreatment (Fig. 2A), consistent with a previous study 16, possibly due to a direct effect of dephosphorylation following GSK470 treatment. Indeed, PDPK1 is responsible for the phosphorylation of many other AGC kinases, such as p70S6K, in addition to Akt 17, 18. Moreover, pretreatment with IGF-1 partially reversed the inhibition of cell proliferation and colony formation (Fig. 2B, C). Immunoblot analysis revealed that the cleavage of Caspase3 and PARP was reduced after pretreatment with IGF-1 (Fig. 2D), suggesting that activating the PI3K-Akt pathway protected against apoptosis induced by GSK470. Collectively, our results suggest that GSK470 targets PDPK1 to suppress the Akt/mTOR pathway, leading to inhibition of cell proliferation and enhanced apoptosis.

### Inhibiting PDPK1 triggers a significant increase in autophagy

**Figure 3:**
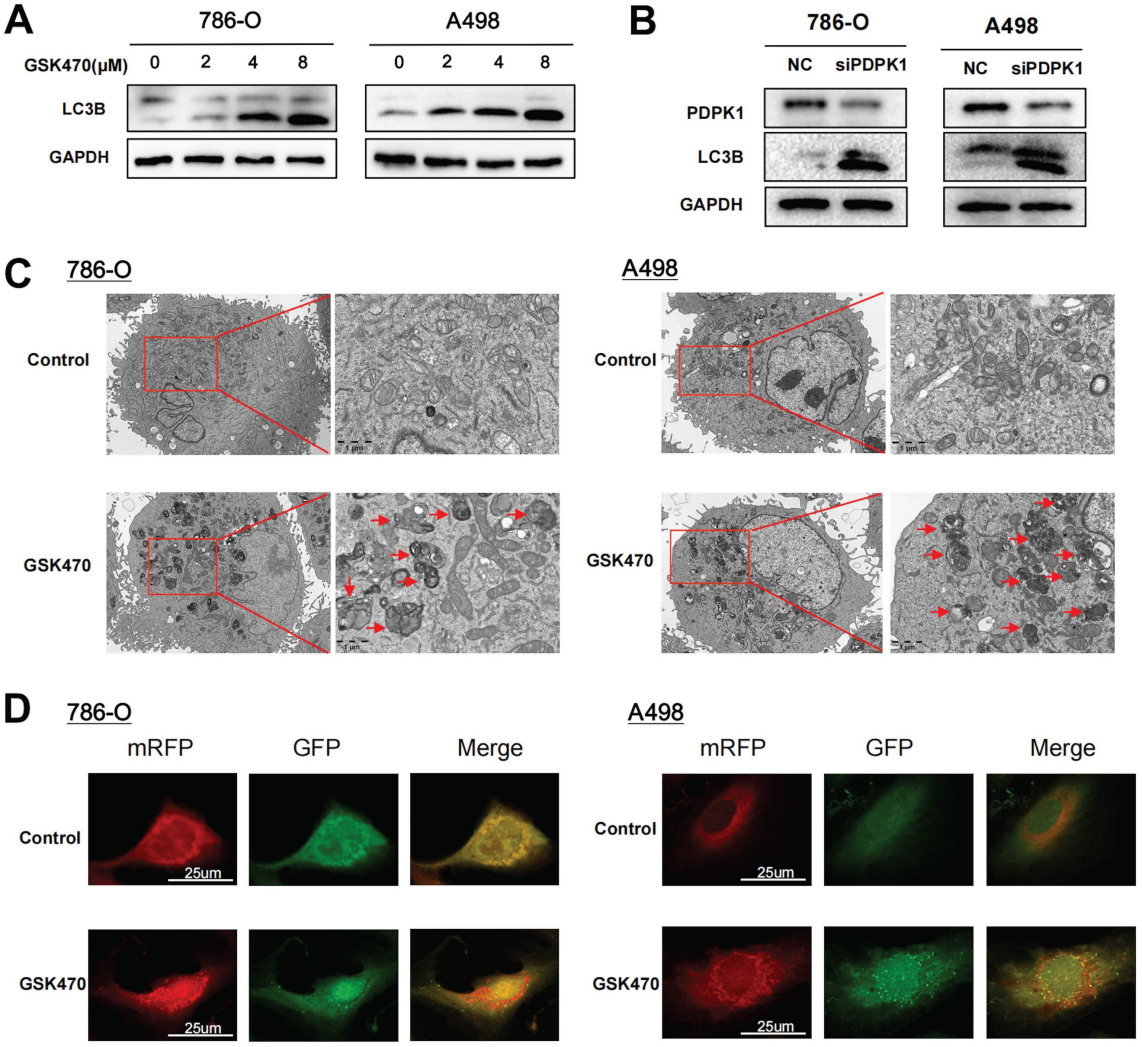
Inhibition of PDPK1 significantly increases autophagy. **A.** Western blotting was performed to assess the expression of the autophagy-related protein LC3 in RCC cells treated with increasing concentrations of GSK470. **B.** Western blot analysis of RCC cells upon siRNA-mediated interference with PDPK1 expression. PDPK1 and LC3 expression was measured. **C.** Autophagosomes were visualized by transmission electron microscopy (6000 ×/20000 × magnification) in RCC cells cultured with GSK470 (4 μM). Scale bar, 1 μm. **D.** RCC cells with stable expression of the mRFP-EGFP-LC3 fusion protein were cocultured with DMSO or GSK470 (4 μM). In the merged images, autophagosomes appear as yellow puncta (RFP+GFP+), while autolysosomes appear as red puncta (RFP+GFP-). Confocal micrographs are shown (2000 × magnification). Scale bar, 25 μm. Mean ± SD, n = 3. * Indicates a significant difference compared with the control group.

mTOR is a well-known initiation factor involved in regulating autophagy. Considering the significant inhibitory effect of GSK470 on mTORC1 phosphorylation, we hypothesized that inhibition of mTOR by PDPK1 blockade is involved in autophagy activation. To test this hypothesis, we cocultured RCC cells with GSK470 or transfected them with a siRNA genetically targeting PDPK1 and compared the expression of the autophagy marker LC3B of these interventions. Immunoblot analyses of A498 and 786-O cells demonstrated that GSK470 promoted a dose-dependent increase in LC3B expression, indicating the activation of autophagy in response to PDPK1 inhibition (Fig. 3A). The same effect was observed in both A498 and 786-O cells upon siRNA-mediated interference with PDPK1 expression (Fig. 3B). Transmission electron microscopy showed a significant increase in the formation of autophagic vesicles in 786-O and A498 cells cocultured with 4 μM GSK470 (Fig. 3C). Furthermore, we established 786-O cells and A498 cells with stable expression of a tandem mRFP-EGFP-LC3 construct to track the stages of the autophagic process. Coculture with 4 μM GSK470 for 24 hours significantly increased the numbers of both yellow (autophagosomes) and red (autolysosomes) puncta, suggesting the induction of autophagic flux in GSK470-treated 786-O and A498 cells (Fig. 3D). Collectively, our data indicate that targeting PDPK1 effectively activates autophagy.

### Inhibition of autophagy synergizes with the antitumor effects of GSK470 *in vitro*

**Figure 4:**
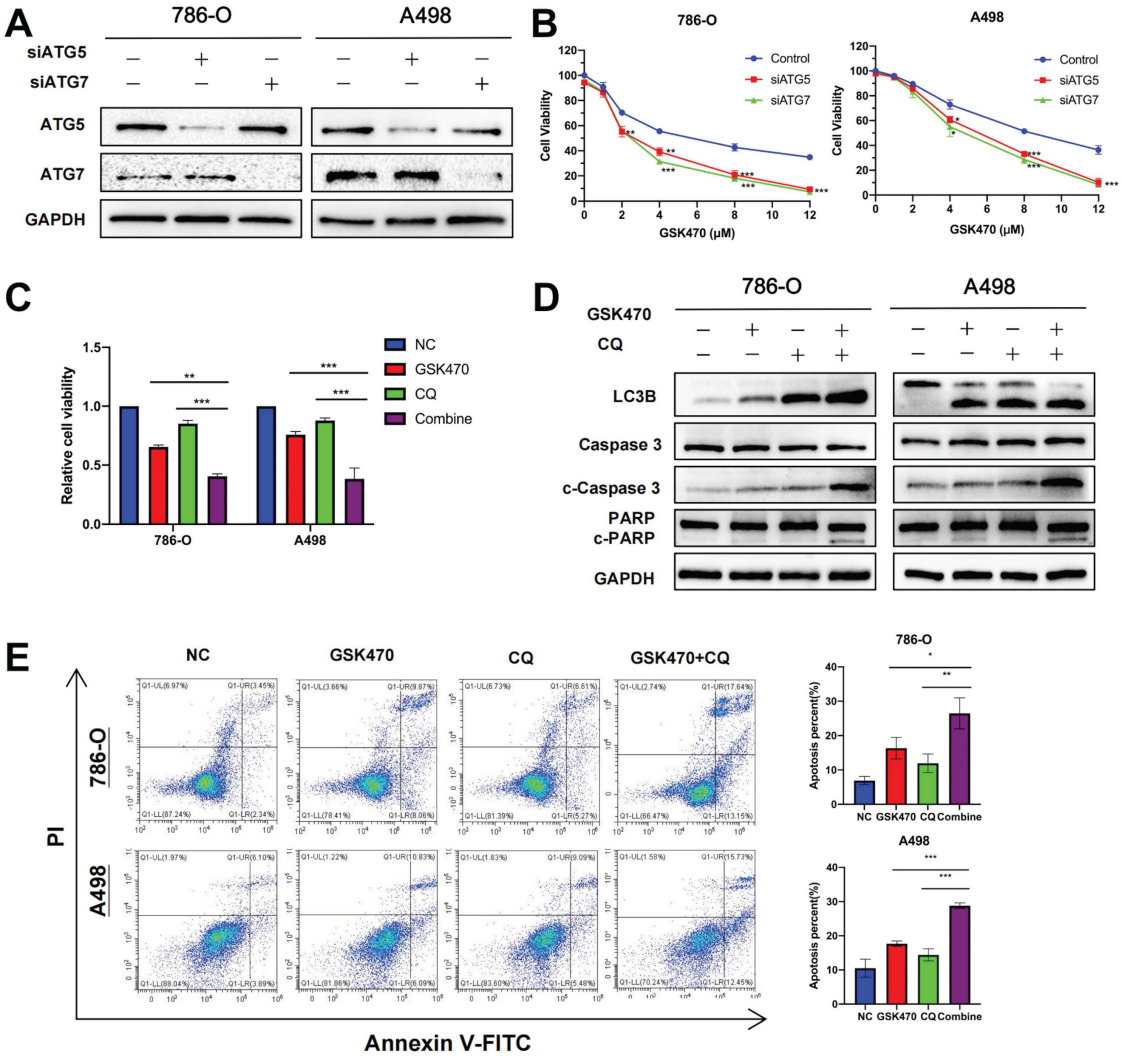
Autophagy inhibition synergizes with the anticancer effects of GSK470.** A.** The autophagy-related genes *ATG5* and *ATG7* were knocked down in 786-O and A498 RCC cells using siRNA. The knockdown efficiency was assessed by immunoblotting. **B.** CCK-8 assay of RCC cells transfected with a nontargeted siRNA as a control or with *ATG5*- or *ATG7*-targeted siRNAs and then treated with the indicated concentrations of GSK470 for 48 h. **C.** CCK-8 assay of RCC cells treated with DMSO, GSK470 (2 μM), CQ (10 μM), or a combination of GSK470 and CQ for 48 h. **D.** Western blot analysis showed that the levels of apoptosis-related proteins were significantly increased upon combined treatment with the autophagy inhibitor CQ and GSK470. RCC cell lines were treated with GSK470 (2 μM), CQ (10 µM), or a combination of GSK470 and CQ for 48 h. **E.** Flow cytometric analysis confirmed the proapoptotic effect of CQ (10 µM) and GSK470 (2 μM) in RCC cell lines after treatment for 48 h. Mean ± SD, n = 3. * indicates a significant difference compared with the control group (or between the indicated groups).

Autophagy acts as a double-edged sword in tumors; excessive autophagy can inhibit tumor growth by eradicating tumor cells, while moderate autophagy can promote cellular self-repair and induce tumor growth 19. Thus, we aimed to determine whether GSK470-induced activation of autophagy is beneficial or detrimental in RCC. Two different genes, *ATG5* 20 and *ATG7* 21, which are essential for functional autophagy, were targeted with siRNAs to block autophagy in 786-O and A498 RCC cells (Fig. 4A). Cells with targeted knockdown of either *ATG5* or *ATG7* exhibited slower proliferation than cells transfected with the nontargeted control siRNA upon treatment with different concentrations of GSK470 (Fig. 4B). This finding suggests that autophagy promotes RCC cell proliferation in the setting of PDPK1 inhibition, confirming that autophagy inhibition may be an effective approach for synergistic treatment of RCC.

CQ has been used for decades to treat malaria, rheumatoid arthritis, and lupus and is an FDA-approved drug that disrupts lysosomal function and consequently inhibits autophagy 22. We next used CQ as a tool to disrupt autophagy. In addition, we selected a relatively low concentration of GSK470 (2 μM; the IC20 in A498 cells) for treatment to accentuate the synergy between this drug and CQ. A498 and 786-O cells were seeded in 96-well or 6-well plates, pretreated with CQ (10 μM) for 4 h and then cocultured with or without GSK470 for 48 h. As expected, the results of the Cell Counting Kit-8 (CCK-8) assay demonstrated that the combined treatment had a greater antiproliferative effect than treatment with either CQ or the inhibitor GSK470 alone (Fig. 4C). The results of immunoblotting showed that the expression of LC3B was increased in the CQ and combined treatment groups (Fig. 4D), confirming that CQ blocked the degradation of LC3B along with other autophagic cargo inside the autophagosome. The levels of the apoptosis-related proteins cleaved Caspase3 (c-Caspase3) and cleaved PARP (c-PARP) were significantly higher after the combined treatment than after treatment with either drug alone (Fig. 4D). Flow cytometric analysis showed that compared with treatment with either drug alone, pretreatment with CQ before GSK470 treatment enhanced the increase in apoptosis in both A498 and 786-O RCC cells (Fig. 4E). The above results indicate that the combined use of an autophagy inhibitor and PDPK1 inhibition may be a useful therapeutic strategy for RCC.

### GSK470 is well tolerated and exhibits enhanced tumor control efficacy when combined with CQ

**Figure 5:**
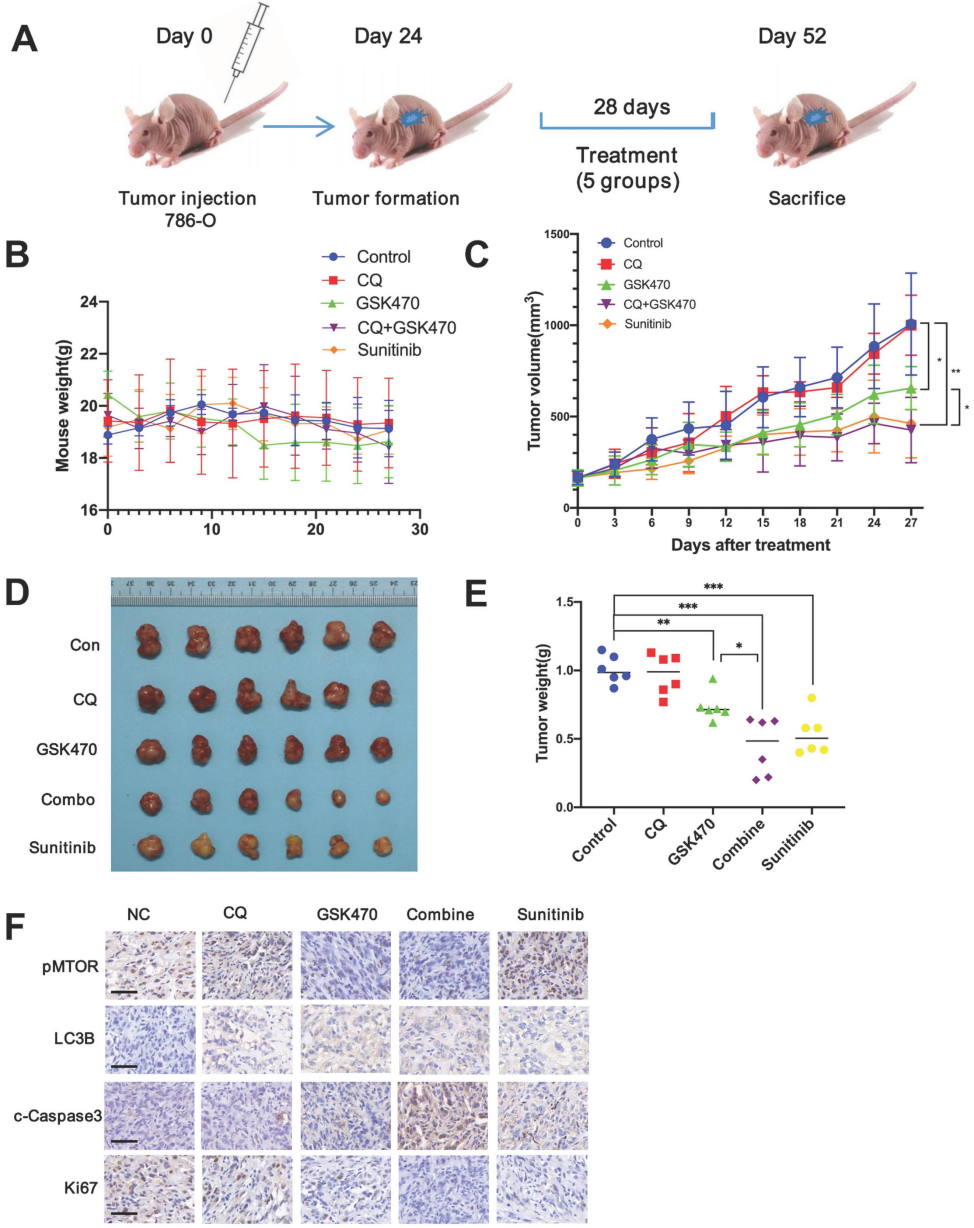
GSK470 inhibits tumor growth in RCC mouse models, and its growth inhibitory effect is enhanced by CQ. **A.** Mice were implanted with 786-O RCC cells, and treatment was initiated on day 24 post-implantation. The mice were treated with (1) vehicle (normal saline, ip, tiw), (2) CQ (65 mg/kg, ip, qd), (3) GSK470 (100 mg/kg, ip, tiw), (4) GSK470+ CQ, or (5) sunitinib (80 mg/kg, po, 5 d/w). Tumor volume and mouse weight were measured in the groups once every three days.** B.** The weight of mice in each group did not differ significantly after treatment. **C.** The tumor volumes in the five groups were recorded after treatment. **D, E** The mice in all five groups were sacrificed, and tumors were then harvested and weighed. **F.** Immunohistochemical staining of tumor specimens. Scale bars = 50 μm. * indicates a significant difference compared with the control group (or between the indicated groups).

We used a 786-O xenograft model of RCC to study the anticancer potential of GSK470 *in vivo*. Sunitinib, a targeted drug commonly used to effectively treat RCC 4, was used as a positive control. The experimental animals were divided into 5 groups: the control, CQ, GSK470, CQ/GSK470 combination, and sunitinib groups (Fig. 5A).

Our results showed that GSK470 was well tolerated, and no notable toxicity was observed after either GSK470 monotherapy or combination therapy with CQ, as evidenced by measurement of body weight, suggesting that GSK470 is safe for administration *in vivo* (Fig. 5B). Treatment with GSK470 resulted in significant inhibition of tumor growth compared to that in the control group (Fig. 5C-E). The combination of GSK470 and CQ exhibited a stronger inhibitory effect on tumor growth than either GSK470 or CQ alone (Fig. 5C-E). Furthermore, the tumor weight did not differ significantly between the combination group and sunitinib group (*P*=0.409), indicating the potent tumor control upon combination treatment with the PDPK1 inhibitor and autophagy inhibitor (Fig. 5C-E). Immunohistochemical analysis of specimens collected from the animals demonstrated that GSK470 significantly reduced the protein levels of p-mTOR and the cell proliferation marker Ki67 but increased those of the autophagic marker LC3B and the apoptosis marker c-Caspase3 (Fig. 5F). The combination CQ/GSK470 treatment group exhibited the lowest Ki67 level and highest c-Caspase level, which were comparable to those in the sunitinib group (Fig. 5F). In summary, collectively, our data demonstrate that GSK470 is orally tolerable, inhibits mTOR activity and induces autophagy *in vivo*, and exhibits significant anticancer efficacy when combined with CQ.

## Discussion

Continuous activation of the PI3K/Akt/mTOR signaling pathway is a typical survival mechanism in human tumor cells 5-7. Thus, many inhibitors that target PI3K, Akt, mTOR and tyrosine kinases have been developed. Sato et al. 8 performed whole-genome, whole-exome, and RNA sequencing of more than 100 patients with RCC and found that multiple PI3K/Akt/mTOR pathway-related mutations were present, in addition to the common VHL gene mutation. Similarly, another study showed that various mutations in PI3K/Akt/mTOR pathway components were detectable in both primary and metastatic lesions of patients with advanced RCC 23. Notably, mutations in the mTOR gene could lead to collective activation of the downstream proteins p70S6K and eIF4, which are believed to be associated with tumor survival and treatment sensitivity 23.

PDPK1 functions downstream of PI3K and is crucial for the activation of Akt and many other AGC kinases 24, 25. Aberrant signaling resulting from PI3K mutations is transduced by PDPK1; thus, targeting PDPK1 has been proposed to reverse the effects of such oncogenic mutations. Exploiting PDPK1 targeting to block aberrant activation of this pathway has attracted increasing attention. Experimental studies have shown that PDPK1 is a potential therapeutic target for breast cancer 26, 27, pancreatic cancer 28, esophageal cancer 29, lymphoma 30, and melanoma 31, 32. Here, we found that inhibition of PDPK1 by GSK470 effectively blocked RCC cell proliferation, increased RCC cell apoptosis and reduced the phosphorylation of Akt at both Thr308 and Ser473, and all of these effects were partially reversed by treatment with the PI3K-Akt pathway activator IGF-1. Our study is consistent with the study by Peter Flynn et al., which revealed simultaneous inhibition of Akt phosphorylation at Thr308 and Ser473 by treatment with PDPK1 antisense oligonucleotides 17. However, other studies found that phosphorylation of only Thr308 was abolished 13, 16. These differences imply that the function of PDPK1 can vary across cellular contexts.

mTOR is a serine/threonine protein kinase and a downstream effector of PI3K and Akt signaling that plays central roles in the regulation of cell proliferation, growth, differentiation, migration and survival 33. Inhibition of mTOR function can lead to inactivation of ribosomal S6K1 and inhibition of cap-dependent translation initiation through the 4E-BP1/eIF4E pathway, thus inducing cell cycle arrest and, potentially, apoptosis 34. As expected, we found that inhibition of PDPK1 by GSK470 suppressed the phosphorylation of mTOR and the downstream mediators p70S6K and 4E-BP1 in RCC cells.

Dysregulation of mTOR signaling has been observed in various human tumors, including RCC, and can render these tumors highly susceptible to mTOR inhibitors 6. Despite the modest benefits of mTOR inhibitors such as everolimus, an FDA-approved drug used to treat metastatic RCC, limited efficacy is observed in some patients. One of the most important factors contributing to drug resistance or insensitivity is therapeutic activation of autophagy, which is induced by mTOR inhibition 35. Autophagy is a cellular process that enables to the degradation and recycling of cellular components, including damaged organelles and unfolded proteins, to maintain cellular homeostasis. Upon mTORC1 inhibition, autophagosomes form, engulfing cytoplasmic proteins and organelles and then fusing with lysosomes, leading to the degradation of cellular components and the recycling of molecular building blocks 36. Autophagy is generally accepted to play dual roles in tumorigenesis, acting as both a tumor suppressor and a protector of cancer cell survival. However, the implications of autophagy activation in RCC remain unclear. Here, we found that disrupting autophagic flux had a substantial anticancer effect on RCC cells treated with the PDPK1 inhibitor GSK470, suggesting that autophagy may have a protective effect that is activated when the PDPK1-Akt-mTOR signaling pathway is blocked.

Studies have shown that combining agents that target mTOR signaling with those that induce autophagy inhibition results in enhanced tumor control compared to that achieved with any single agent in multiple cancer types 37-39. A phase I/II trial evaluating everolimus and hydroxychloroquine in patients with RCC already treated with 1-3 regimens showed good tolerability and efficacy, with a 6-month PFS rate of >40%. Herein, our preliminary results showed a synergistic effect on cell death in RCC cell lines and a mouse model upon combination treatment with GSK470 and CQ, indicating that inhibiting autophagy increases the sensitivity of RCC to PDPK1 inhibitors. Thus, our research establishes the combined use of PDPK1 inhibitors and autophagy inhibitors as a novel strategy for RCC treatment, but further investigation to establish the safety and efficacy of this strategy is needed.

## Conclusion

**Figure 6:**
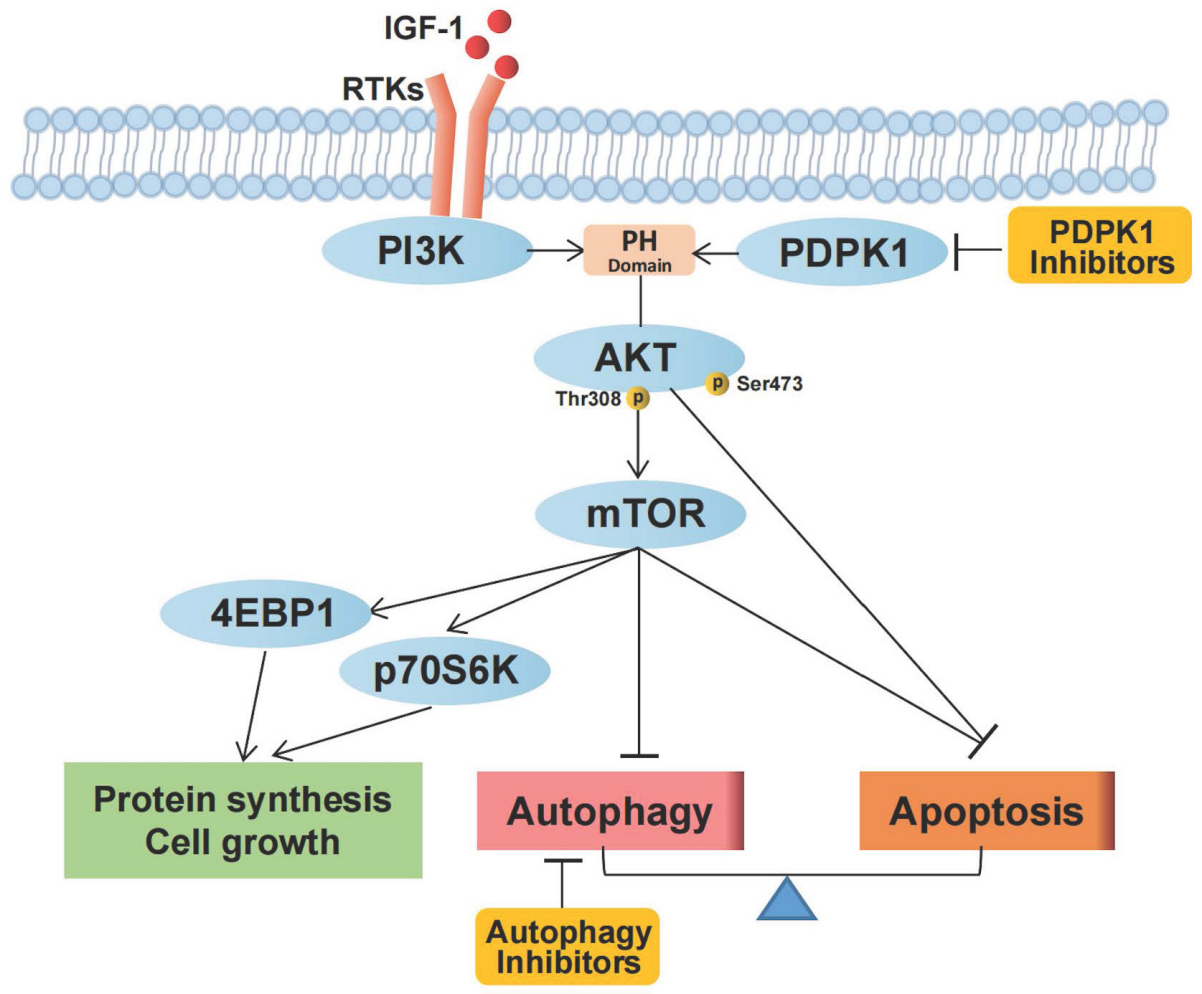
Schematic representation of the effects of PDPK1 on the PI3K-Akt-mTOR pathway and autophagy resulting in regulation of proliferation and apoptosis in RCC.

Our study confirms that targeting PDPK1 inhibits the phosphorylation of Akt1 and, through a cascade of downstream effects, suppresses mTORC1 activity, which is related to protein synthesis, cell growth, apoptosis, and autophagy in RCC. Autophagy plays a protective role in the setting of PDPK1 inhibition, and combined targeting of PDPK1 and autophagy synergistically inhibits RCC pathogenesis (Fig. 6). However, our study has some limitations. PDPK1 functions downstream of PI3K and plays a crucial role in the activation of Akt and several other AGC kinases, including PKC, S6K, SGK, and RSK 7, 17. Moreover, PDPK1 is involved in the Ras/MAPK and Myc pathways, and inhibiting PDPK1 may lead to inhibition of mTORC1 through alternative pathways such as the PDPK1-SGK1 signaling pathway 40. Therefore, PDPK1 can be considered to target both Akt and molecules other than Akt, and it is essential to fully understand its role in RCC.

## Figures and Tables

**Table 1:** The primer sequences used of this study.

Primer sequences		
GAPDH	Forward	GGTGTGAACCATGAGAAGTATGA
	Reverse	GAGTCCTTCCACGATACCAAAG
AGT5	Forward	AGAAGCTGTTTCGTCCTGTGG
	Reverse	AGGTGTTTCCAACATTGGCTC
ATG7	Forward	GGTGTGAATGCCAGAGGATT
	Reverse	CCATCAATAGGAAGACGACATCAT
PDPK1	Forward	ACCAGCCAGCTGTATGACG
	Reverse	GTCCTGCCACAAGCTGGTAT

## Abbreviations

RCC: Renal cell carcinoma
PDPK1: 3-Phosphoinositide-dependent kinase 1
GSK470: GSK2334470
IGF-1: insulin-like growth factor-1
mTORC1: mTOR complex 1
ccRCC: clear cell renal carcinoma
PFS: progression-free survival
TKI: tyrosine kinase inhibitors
CQ: chloroquine
CCK-8: Cell Counting Kit-8
PARP: poly ADP-ribose polymerase
